# Long-term woodland restoration on lowland farmland through passive rewilding

**DOI:** 10.1371/journal.pone.0252466

**Published:** 2021-06-16

**Authors:** Richard K. Broughton, James M. Bullock, Charles George, Ross A. Hill, Shelley A. Hinsley, Marta Maziarz, Markus Melin, J. Owen Mountford, Tim H. Sparks, Richard F. Pywell

**Affiliations:** 1 UK Centre for Ecology & Hydrology, Wallingford, Oxfordshire, United Kingdom; 2 Department of Life and Environmental Sciences, Bournemouth University, Talbot Campus, Poole, Dorset, United Kingdom; 3 Museum and Institute of Zoology, Polish Academy of Sciences, Warsaw, Poland; 4 Natural Resources Institute Finland, Joensuu, Finland; 5 Institute of Zoology, Poznań University of Life Sciences, Poznań, Poland; 6 Museum of Zoology, University of Cambridge, Cambridge, United Kingdom; Technical University in Zvolen, SLOVAKIA

## Abstract

Natural succession of vegetation on abandoned farmland provides opportunities for passive rewilding to re-establish native woodlands, but in Western Europe the patterns and outcomes of vegetation colonisation are poorly known. We combine time series of field surveys and remote sensing (lidar and photogrammetry) to study woodland development on two farmland fields in England over 24 and 59 years respectively: the New Wilderness (2.1 ha) abandoned in 1996, and the Old Wilderness (3.9 ha) abandoned in 1961, both adjacent to ancient woodland. Woody vegetation colonisation of the New Wilderness was rapid, with 86% vegetation cover averaging 2.9 m tall after 23 years post-abandonment. The Old Wilderness had 100% woody cover averaging 13.1 m tall after 53 years, with an overstorey tree-canopy (≥ 8 m tall) covering 91%. By this stage, the structural characteristics of the Old Wilderness were approaching those of neighbouring ancient woodlands. The woody species composition of both Wildernesses differed from ancient woodland, being dominated by animal-dispersed pedunculate oak *Quercus robur* and berry-bearing shrubs. Tree colonisation was spatially clustered, with wind-dispersed common ash *Fraxinus excelsior* mostly occurring near seed sources in adjacent woodland and hedgerows, and clusters of oaks probably resulting from acorn hoarding by birds and rodents. After 24 years the density of live trees in the New Wilderness was 132/ha (57% oak), with 390/ha (52% oak) in the Old Wilderness after 59 years; deadwood accounted for 8% of tree stems in the former and 14% in the latter. Passive rewilding of these ‘Wilderness’ sites shows that closed-canopy woodland readily re-established on abandoned farmland close to existing woodland, it was resilient to the presence of herbivores and variable weather, and approached the height structure of older woods within approximately 50 years. This study provides valuable long-term reference data in temperate Europe, helping to inform predictions of the potential outcomes of widespread abandonment of agricultural land in this region.

## Introduction

The destruction and fragmentation of forests through expansion of industry, urbanisation and agriculture is a major driver of global biodiversity loss [1]. Those regions with a long history of industrialisation and agricultural intensification have a greater legacy of deforestation, including Western Europe [2, 3]. In these deforested regions, remaining woodland tends to be highly fragmented within an agricultural landscape, representing a challenge for those specialist forest species with requirements for extensive habitat but limited dispersal capabilities [4–7].

Whilst deforestation remains a major concern in tropical regions, in many parts of Europe there has been a recent trend towards increasing woodland cover [8, 9]. Planned afforestation and woodland creation has focused on tree-planting schemes, but there has also been widespread abandonment of agricultural land, and its natural colonisation by shrubs and woodland, which is receiving interest as an alternative means of afforestation and ecosystem restoration [10–14].

Natural regeneration of secondary woodland commonly occurs on abandoned fields that were originally deforested and cleared for agriculture in previous centuries; this process has been well studied in North America [15–18], but less so in Europe [16, 17]. Examples of re-colonisation of former farmland by tree cover are also found worldwide, and this can be the basis of native woodland restoration through so-called ‘passive rewilding’ [19].

### Passive rewilding for woodland restoration

The concept of rewilding has evolved to mean the restoration of self-regulating and dynamic ecosystems free from direct human interference, while also considering stakeholder needs and perceptions of ‘wildness’ in society [20]. ‘Passive’ rewilding refers to spontaneous development of ecosystems without direct intervention, such as on abandoned land [16, 20] and occurs via natural succession. Under this rewilding framework, restored woodland ecosystems on former farmland would generate self-regulating processes of vegetation development and disturbance regimes, trophic complexity, and species colonisations via unimpeded dispersal, providing valuable ecosystem services for society [19, 21].

As a means of woodland restoration on abandoned land, passive rewilding has several advantages over planting, including preservation of local plant genotypes, avoiding the introduction of pests and diseases on imported saplings, better resilience to drought during establishment, and reduced management costs [22, 23]. However, while there is a relatively large ‘old field’ literature for the outcomes of passive rewilding of abandoned farmland in North America [17], and some for Northern Europe [24–26], relatively little attention has been paid to intensively farmed landscapes in Western or Central Europe [12, 16]. This sparse evidence for much of Europe is despite the fact that agricultural land abandonment is currently one of the major land use changes in the region [9].

The natural succession of woodland on abandoned land may have mixed impacts, as many farmland plants and animals will be replaced by forest species over time [27]. The limited information from Europe indicates that such processes have reduced important open habitats in mountainous regions [10], but can benefit some lowland bird, butterfly and plant communities [14, 28, 29]. Additionally, small patches of restored woodland or early successional shrubland can diversify landscapes dominated by agriculture, supporting a wider variety of species overall [30, 31], and providing important habitat refuges for some [32]. Natural expansion of existing woodlands onto adjacent farmland can also buffer mature forest from edge effects and the influence of agriculture [33, 34].

How and why abandoned farmland becomes colonised by woodland has important effects on the wooded habitats that develop, including the species composition, trajectories of habitat succession and the biodiversity that they support. Such outcomes also have important implications for both planned and unplanned woodland restoration through passive rewilding. By understanding the patterns and dynamics of woodland colonisation of farmland under different scenarios, useful projections can be made for the outcomes over varying timescales [12, 19].

Comprehensive literature reviews of ‘old field’ succession, predominantly from North America but also less common examples worldwide [16, 17], summarised that the trajectories of vegetation colonisation mostly depend on climate, past land use, soil type and the surrounding vegetation community. These syntheses also suggest that, in benign climates such as temperate Europe, colonisation and succession should be dominated by biotic factors, particularly by local tree species and their ability to disperse and colonise. However, long-term evidence from Europe to confirm these assumptions is critically lacking. Building the knowledge base of the timescales, trajectories and composition of woodland succession on abandoned European farmland is therefore important to understand what challenges and opportunities may arise from the widespread land abandonment [9].

### Monitoring woodland regeneration

Woodland restoration and natural regeneration studies commonly rely on chronosequences, which substitute space-for-time to compare contemporary sites at different stages of development [16, 17]. However, chronosequences can give misleading representations of vegetation succession if sites and timescales are inappropriate [35, 36]. Long-term monitoring of individual site development can provide more direct and definitive information on the trajectory of ecosystem development over time, but such studies are rare [36].

In Europe, the best examples of direct observation of woodland regeneration on farmland come from long-term monitoring sites in southern England. Natural succession of woodland on two small (0.26–1 ha) former arable plots have been recorded over more than a century at the Broadbalk and Geescroft ‘Wilderness’ sites in Hertfordshire [37]. ‘Wilderness’ is the term originally given to these monitored reference sites on abandoned plots, reflecting the thicket of vegetation that initially develops. A larger (4 ha) Wilderness site at Monks Wood in Cambridgeshire was established as a reference site for natural succession, and has been partially documented over its initial 38 years of development from an arable field to secondary woodland [38]. A second monitored Wilderness (2.5 ha) was later established as an additional reference site on grassland at Monks Wood, with development reported for the initial 6 years post-abandonment [39].

These Wilderness studies indicate that abandoned farmland in temperate Western Europe can be colonised by native woodland plants within just a few years, which is also typical of passive restoration of tropical and temperate forests, such as the ‘old fields’ in eastern North America [16, 17]. However, the species composition on restored sites can differ from that of long-established woodland due to differential dispersal, colonisation abilities and seedling mortality among trees and shrubs [16, 40, 41]. Initial site conditions, such as disturbed ground or dense grassy sward, shading, competition and proximity to nearby seed sources also have strong effects on species composition and plant densities [15–18, 37].

Whilst studies of woodland regeneration on abandoned farmland have largely focused on vegetation composition, less attention has been paid to the spatial patterns and structural characteristics of the developing thickets and tree canopy [14, 37, 39]. Such information is important for mapping and predicting the types and extent of habitat that may develop over certain timescales or calculating carbon storage [42–44]. However, there is a challenge in characterising complex three-dimensional woody vegetation in sufficient detail and extent using standard ground-based surveys.

The increasing availability of remote sensing data can overcome these limitations to assess woodland coverage and structure at unprecedented extents and resolution [45]. In particular, airborne lidar (light detection and ranging) can map entire forests at resolutions of sub-1 m, enabling detailed inventories of woodland canopy and understorey structure [46, 47]. Such information has been used to describe species-habitat associations between forest fauna and vegetation [48, 49]. Remote sensing therefore offers increasing opportunities to complement field surveys in assessing woodland restoration on abandoned land.

### Study objectives

In this study, we revisit the two Wilderness reference sites at Monks Wood to assess the secondary woodland restoration by passive rewilding of abandoned farmland. We use long-term monitoring data from field surveys and remote sensing (primarily lidar) to quantify the composition and spatial patterns of woodland development over time, for up to 59 years post-abandonment. We also compare the developing vegetation with other local ancient woodland sites, to assess the relative structural maturity and composition. Based on general principles of woodland regeneration [16, 17], we hypothesise that there should be rapid colonisation of the two Wildernesses at Monks Wood by woody vegetation. We also expect the species composition on the sites to differ from the neighbouring mature woodlands, likely reflecting the dispersal and establishment capabilities of local trees and shrubs [17, 50]. Associated with the dispersal mode and seed source availability, we also test whether tree colonisation is clumped or evenly distributed, and whether this results in patchy regeneration across the sites. In essence, our overriding research questions are to ask what type of woodland composition develops on the sites, and if woodland coverage is patchy or complete closed-canopy across the sites, and how long does it take for closed-canopy woodland to develop.

This study is the first to demonstrate the monitoring of natural succession using time series of lidar remote sensing data, and how this direct observation can quantify woodland restoration on former farmland. We discuss the observed patterns of woodland restoration and its potential to support afforestation and rewilding policies that aim to enhance biodiversity and provide wider ecosystem services. The study provides valuable direct evidence from long-term reference sites in temperate Europe, helping to inform predictions of the potential outcomes of widespread abandonment of agricultural land in this region.

## Materials and methods

### Study area

**Fig 1:**
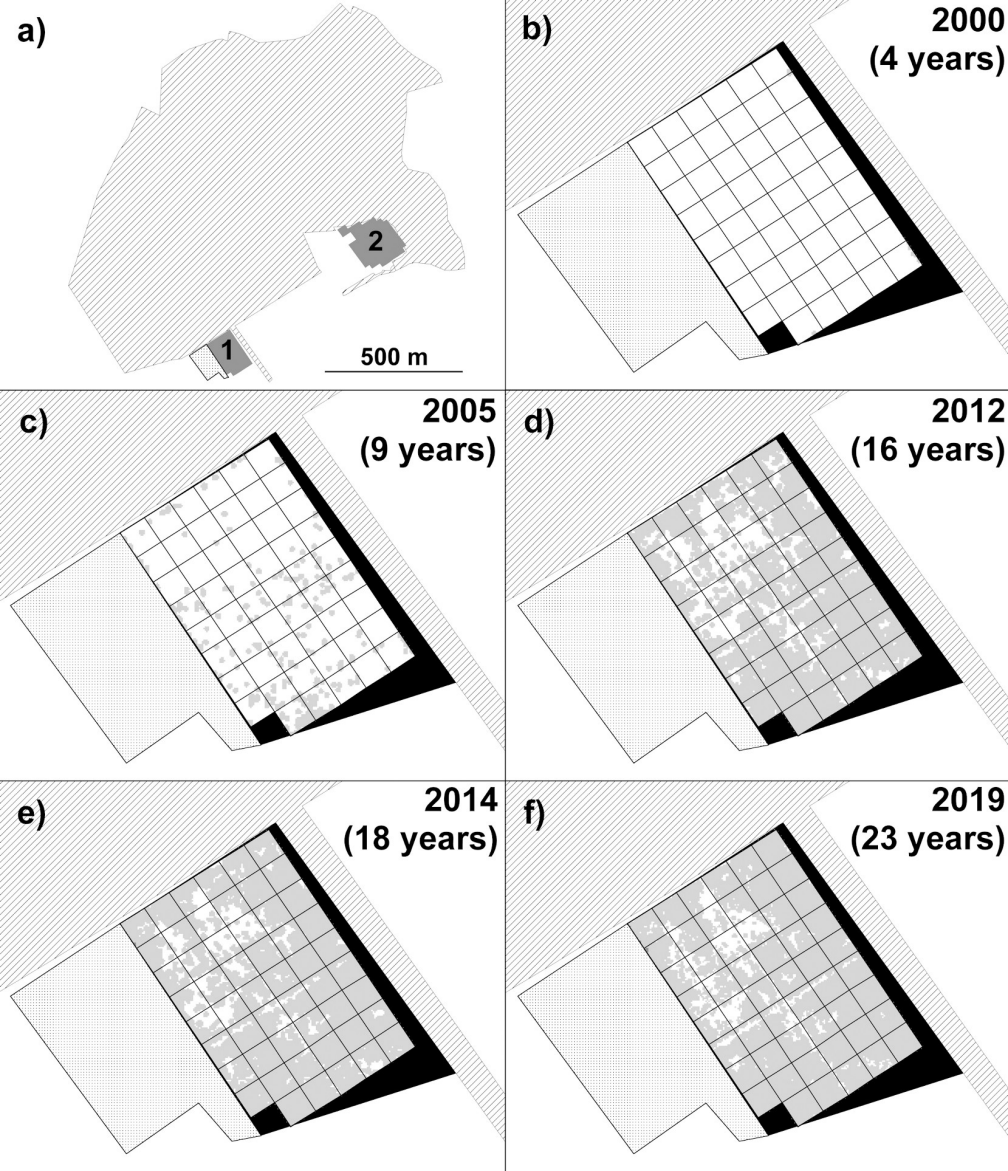
Study sites and New Wilderness development. Wilderness study sites in relation to Monks Wood ancient woodland and associated mature hedgerow (hatched area) and block of unstudied scrub (stippled area): a) location of the New Wilderness study area (dark grey block marked ‘1’) and Old Wilderness (dark grey block marked ‘2’); Panels b) to f): Time series of remote sensing (lidar 2000–2014, structure-from-motion photogrammetry 2019) data for the New Wilderness for data collected in a given year (and number of years since Wilderness abandonment in 1996). Pale grey areas denote extent of woody vegetation of ≥ 0.5 m but below 8 m tall. White areas are grass/herb vegetation below 0.5 m tall. Black areas denote unstudied parts of the New Wilderness area. Grid cells are 20 x 20 m.

The study focussed on the two Wilderness reference sites of abandoned agricultural fields adjacent to the 157 ha Monks Wood National Nature Reserve in Cambridgeshire, eastern England (52° 24’N, 0° 14’W; Fig 1). Monks Wood is a semi-natural ancient woodland (existing since at least 1600 AD, [22]), dominated by common ash *Fraxinus excelsior*, pedunculate oak *Quercus robur* and field maple *Acer campestre* in the overstorey tree canopy, with some silver birch *Betula pendula*, European aspen *Populus tremula*, elm *Ulmus* spp., wild service *Sorbus torminalis*, and willows *Salix cinerea* and *S*. *caprea*. The understorey is dominated by hawthorn *Crataegus* spp., blackthorn *Prunus spinosa* and common hazel *Corylus avellana*, with some common dogwood *Cornus sanguinea*, wild privet *Ligustrum vulgare*, crab apple *Malus sylvestris*, rose *Rosa canina* and *R*. *arvensis*, and bramble *Rubus fruticosus* [51, 52]. Much of Monks Wood was clear-felled around 1918–20, but leaving the stumps and soils intact, and has since regrown with negligible management across ~90% of its area, except for ride and glade maintenance.

Two agricultural fields on the southern edge of Monks Wood were abandoned in the 20^th^ Century. The 4.0 ha Stocking Close field was probably cleared of woodland in the Roman period, and was cultivated between at least 1850 and a final crop of barley in 1960. After ploughing in autumn 1961, the field was abandoned to passive rewilding [53] with no management except for annual clearance of a circular path (~1.5 m wide) which ceased around 2008. The field was renamed the ‘Monks Wood Wilderness’ [54], hereafter the ‘Old Wilderness’.

Further woodland clearance between 1612 and 1820 included the 2.5 ha Com’s Field [51], which was under cultivation and livestock grazing during the 19-20^th^ Centuries, later being maintained as unimproved grassland from at least 1970. In summer 1996 the field was mown for a final time and abandoned as a second Wilderness site, except for annual mowing of paths, and renamed as ‘Wilderness 2’ [39], hereafter the ‘New Wilderness’. In 1998, to aid recording, both Wildernesses were compartmentalised into approximate 20 × 20 m grid cells, marked with numbered posts at intersections.

**Fig 2:**
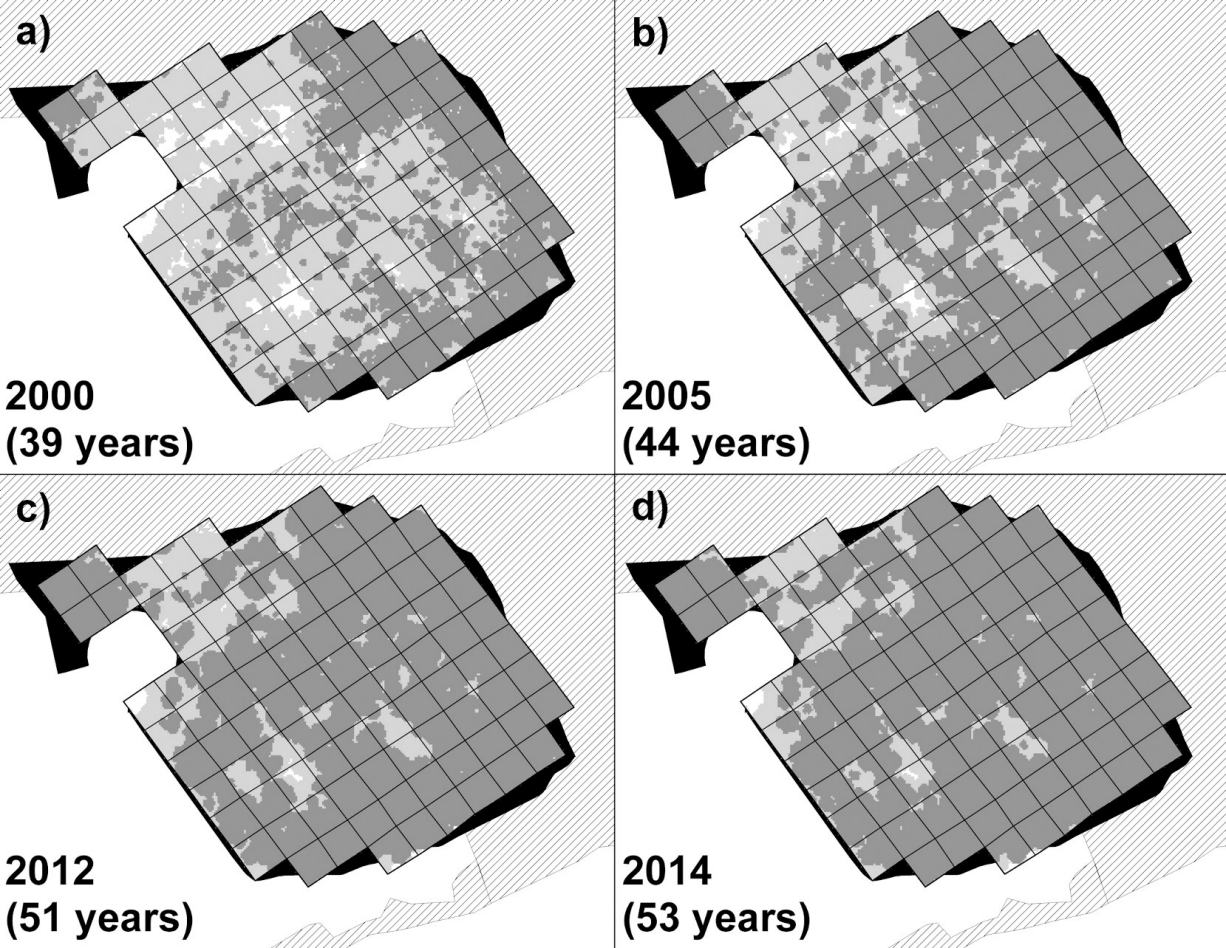
Development of the Old Wilderness vegetation. a) to d): Time series of remote sensing (lidar) data for the Old Wilderness for data collected in a given year (and number of years since Wilderness abandonment in 1961). Pale grey areas denote extent of woody vegetation of ≥ 0.5 m but below 8 m tall, dark grey areas denote vegetation ≥ 8 m tall (overstorey tree canopy). White areas are grass/herb vegetation below 0.5 m tall. Black areas denote unstudied parts of the Old Wilderness area. Hatched areas are Monks Wood ancient woodland and associated mature hedgerow (see Fig 1). Grid cells are 20 x 20 m.

Both Wildernesses lie on moderately well-drained clay soils, on slightly sloping land of c. 35–40 m elevation [51]. The Old Wilderness is surrounded by Monks Wood on two sides and mature trees on a third, with a complex of buildings and open landscaping to the west. The New Wilderness lies 630 m west of the Old Wilderness, with Monks Wood to the north and a ‘green lane’ of hedgerows and mature trees (common ash and pedunculate oak) to the east, with scrub to the west and a hedge and arable farmland to the south (Figs 1 and 2). Monks Wood and the mature hedgerow trees were considered as seed sources for colonising woody plants.

### Remote sensing of vegetation height and cover

The vegetation over the entire area of both Wildernesses was surveyed using remote sensing. A time series of airborne lidar data depicting vegetation heights and spatial cover were available for both Wildernesses during leaf-on (summer) conditions in 2000, 2005, 2012 and 2014 (S1 Table). The 2014 lidar data also included the Monks Wood woodland and eleven other mature ancient woodlands within 5 km of the study area, of a similar woody composition to Monks Wood, for comparison with the Wildernesses. The extent of each woodland was derived from the 2015 National Forest Inventory for England [55] GIS data.

The lidar data were acquired using a sensor mounted on a fixed-wing aircraft at 1–2.1 km altitude, which detects the elevation of the vegetation surface below by scanning the landscape with laser pulses [47]. The data were processed by extracting the first or only surface returns of each laser pulse, attainable from every dataset, which were classified into ground or vegetation returns following standard methods [56]. A canopy height model (CHM) was generated for each coverage by subtracting a digital terrain model (DTM) depicting the ground elevation from the digital surface model (DSM) of above-ground lidar values depicting vegetation heights. The DTM originated from a further data acquisition during leaf-off conditions in March 2014 (S1 Appendix), to detect the terrain more accurately, and was interpolated at 1 m^2^ resolution from classified ground hits using inverse distance weighted interpolation. The resulting CHMs depicted the vegetation development over the time series at a comparable 1 m^2^ horizontal resolution and 0.01 m vertical resolution, although data accuracy resulted in values reported to 0.1 m. Further details of the processing and parameters of different sensors used in the data acquisitions are given in the S1 Appendix.

An additional coverage of the entire New Wilderness was acquired for summer leaf-on conditions in June 2019 (S1 Table) using a Mavic 2 Pro (DJI, Shenzhen, China) unmanned aerial vehicle (UAV, or ‘drone’). The UAV had an on-board 20 megapixel (5472 x 3648 pixels) Hasselblad RGB camera, and 567 images were acquired under high-altitude light cloud conditions, resulting in very few dark shadows. The images were stitched together using ‘structure from motion’ (SfM) software (Agisoft Metashape version 1.6.3 build 10732, Agisoft LLC, St Petersburg, Russian Federation) resulting in a DSM of 11 x 11 mm pixels. The coverage was reprocessed to a 1 m^2^ resolution CHM by subtracting the lidar-derived DTM, and elevations were calibrated and corrected by matching height values to areas of permanent open ground in the lidar data. The lidar and UAV remote sensing for the New Wilderness were therefore available for five coverages between four and 23 years after abandonment (Fig 1), and for the Old Wilderness from four coverages between 39 and 53 years after abandonment (Fig 2). The original CHM datasets for the Wilderness study areas and nearby woods are available as S1 Datasets.

Due to surrounding tree canopies partially overshading some margins of both Wildernesses, only those 20 m grid cells that were unobscured from above were included in analyses, giving 95 cells for the Old Wilderness and 53 cells for the New Wilderness.

Values in the CHMs below 0.5 m were discarded to exclude ground foliage of grasses/herbs, with taller height values considered as woody vegetation of shrubs and trees. The mean height and standard deviation of woody vegetation pixels were extracted for each Wilderness survey area from each of the lidar and photogrammetric coverages in the time series, and also for Monks Wood and other local woods in the 2014 lidar data. The proportion of pixels with height values of 0.5 m or above was taken as the extent of woody cover in each Wilderness or wood. The proportion of pixels with height values of 8 m or above were taken as the extent of the overstorey canopy, reflecting mature or semi-mature tree cover. This 8 m threshold was based on previous assessment of the structure of Monks Wood [46].

### Field surveys of vegetation

Data from various historic and current field surveys of the vegetation in the Wildernesses and Monks Wood were analysed, accounting for variation in methods (S1 Table).

In both Wildernesses, measurements of all trees were undertaken across the entire area of each site, at up to five points in the succession, depending on vegetation development and resources. New Wilderness trees were surveyed first in 2020, once some trees had reached a sufficient size, and access through the growing thicket became possible. Tree species were counted into dbh (diameter at breast height, i.e. 1.3 m) size classes, recorded as living or dead (including fallen deadwood), and assigned to each grid cell. The dbh size classes were 3.2–9.9 cm, 10–29.9 cm and ≥ 30 cm. The minimum dbh for recording (3.2 cm) corresponded to a girth of 10 cm for a circular trunk and dividing this value by 3.14 (π).

New Wilderness shrubs were sampled in 2019. Unlike the total survey coverage of trees, shrub species occurrence was assessed by sampling, due to the impracticality of field surveys of the thickets across the entire site. Instead, shrub occurrence was calculated as the percentage of 1 m^2^ quadrats at 32 of the 20 m grid cell intersections in which each species was detected as present (growing in or over the quadrat). Tree and shrub data for the New Wilderness are available as S2 Dataset.

The Old Wilderness trees were surveyed for the first time in 1998 to record, mark with numbered tags and measure the girth at breast height (1.3 m) of all pedunculate oak, field maple and common ash trees with a girth of at least 10 cm. Girths were converted to dbh by assuming a circular trunk (see above), and assigned to the three size classes as per New Wilderness trees. The Old Wilderness trees were assigned to a grid cell, and repeat surveys were carried out in 2002, 2008, 2013 and 2018, when previously marked trees were also recorded as living or dead (including as fallen deadwood). A 2020 survey recorded other tree species using the same protocol.

The Old Wilderness shrubs were surveyed in 2015. As with the New Wilderness, field surveys of shrubs over the total area were impractical. Instead, shrub species occurrence in the Old Wilderness was recorded in 1 m^2^ quadrats at 91 cell intersections, and calculated as percentage frequency occurrence, as per shrub sampling in the New Wilderness. Tree and shrub data for the Old Wilderness are available as S3 Dataset.

Monks Wood field survey data were available from 2006, from 33 sample transects measuring 100 × 10 m (0.1 ha) in unmanaged stands [57]. Transects were widely distributed across the wood and considered representative, being derived from surveys of bird habitat [58]. Trees were counted in dbh size classes of < 10 cm, 10–29.9 cm and ≥ 30 cm and recorded as living or dead (including fallen deadwood). For the < 10 cm dbh class, the minimum recordable tree of 1.3 m tall was not comparable with the Wilderness surveys (which used a minimum 3.2 cm diameter at 1.3 m tall), although the larger categories were compatible. Shrub species occurrence in Monks Wood was calculated as percentage occurrence in transect samples, as per the quadrat samples in the Wilderness surveys. Tree and shrub data for Monks Wood are available as S4 Dataset.

Comparison between surveys carried the caveat that the total sampled area of Monks Wood (3.3 ha) represented 2% of the woodland area, whereas the sampled area of both Wildernesses (32 m^2^ and 91 m^2^) was 0.2% of each area. However, the greater survey area in Monks Wood reflected the larger scale of the wood due to its greater maturity and size (157 ha) compared with the Wildernesses (2.1 and 3.9 ha under analysis).

### Statistical methods

To test for clustering or patchy distribution of tree regeneration, we used the Global Moran’s I tool in ArcGIS [59]. This tool tested for spatial autocorrelation in the distribution of trees in both Wildernesses, assessing clustering of total tree counts (all species combined) in grid cells versus the null hypothesis of a random or even distribution across cells. The Moran’s I test [60] used an inverse distance method of assessing tree counts between cells, with no distance threshold, and row standardised weighting of counts to allow for aggregation within cells [59]. Total tree counts for the Old Wilderness used 2018–20 survey data, and the New Wilderness used the 2020 data.

To further test for uneven tree regeneration resulting from the distance from seed sources, we used zero-inflated negative binomial models (ZINB) and the pscl package in R [61]. These models tested differences in the distributions of the number of the two dominant trees (pedunculate oak and common ash) in both Wildernesses in relation to the distance from seed sources in surrounding woodland or mature trees. We expected that the wind-dispersed seeds of common ash would result in decreasing abundance with increasing distance from seed sources, whereas the animal-dispersed seeds of pedunculate oak would have a more even distribution among grid cells.

We generated separate ZINB models for oaks and ashes that included their counts (live and dead) in grid cells as the response variables, and the covariate was the distance from the centre of each 20 m grid cell to the adjacent Monks Wood or hedgerow. We used the most recent tree data from 2018 for the Old Wilderness and 2020 for the New Wilderness. To account for potential spatial autocorrelation of tree counts in the grid cells, we first tested the importance of the random effect of the cell identity using generalised least-squares estimation models (GLS), and comparing a null model with one containing only a random factor of the cell number. As this random effect was insignificant, we did not include cell identity in the models.

## Results

### Vegetation height and cover

**Fig 3:**
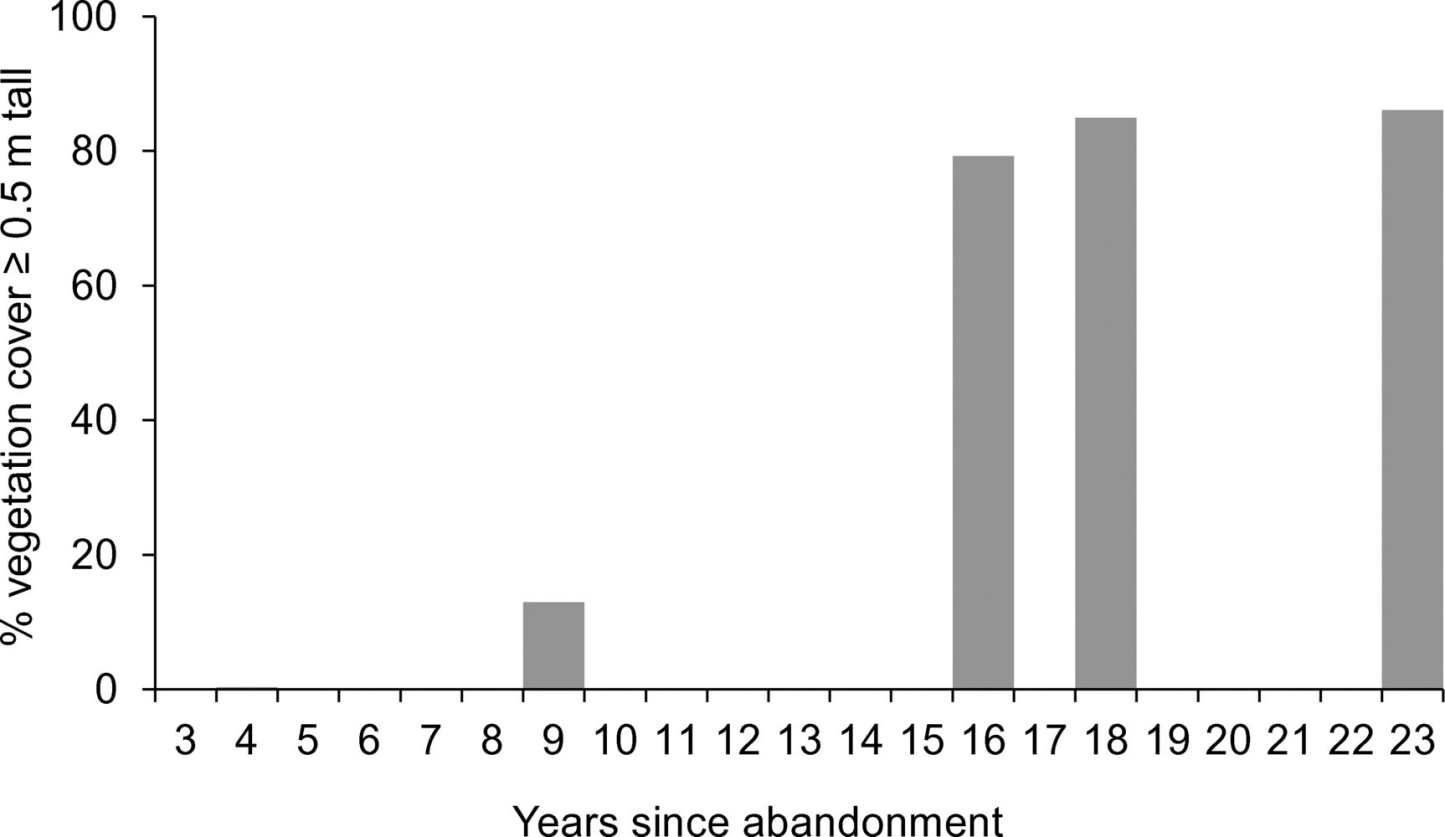
New Wilderness vegetation cover. Progressive coverage of vegetation of at least 0.5 m tall in the New Wilderness in the years since abandonment in 1996.

**Fig 4:**
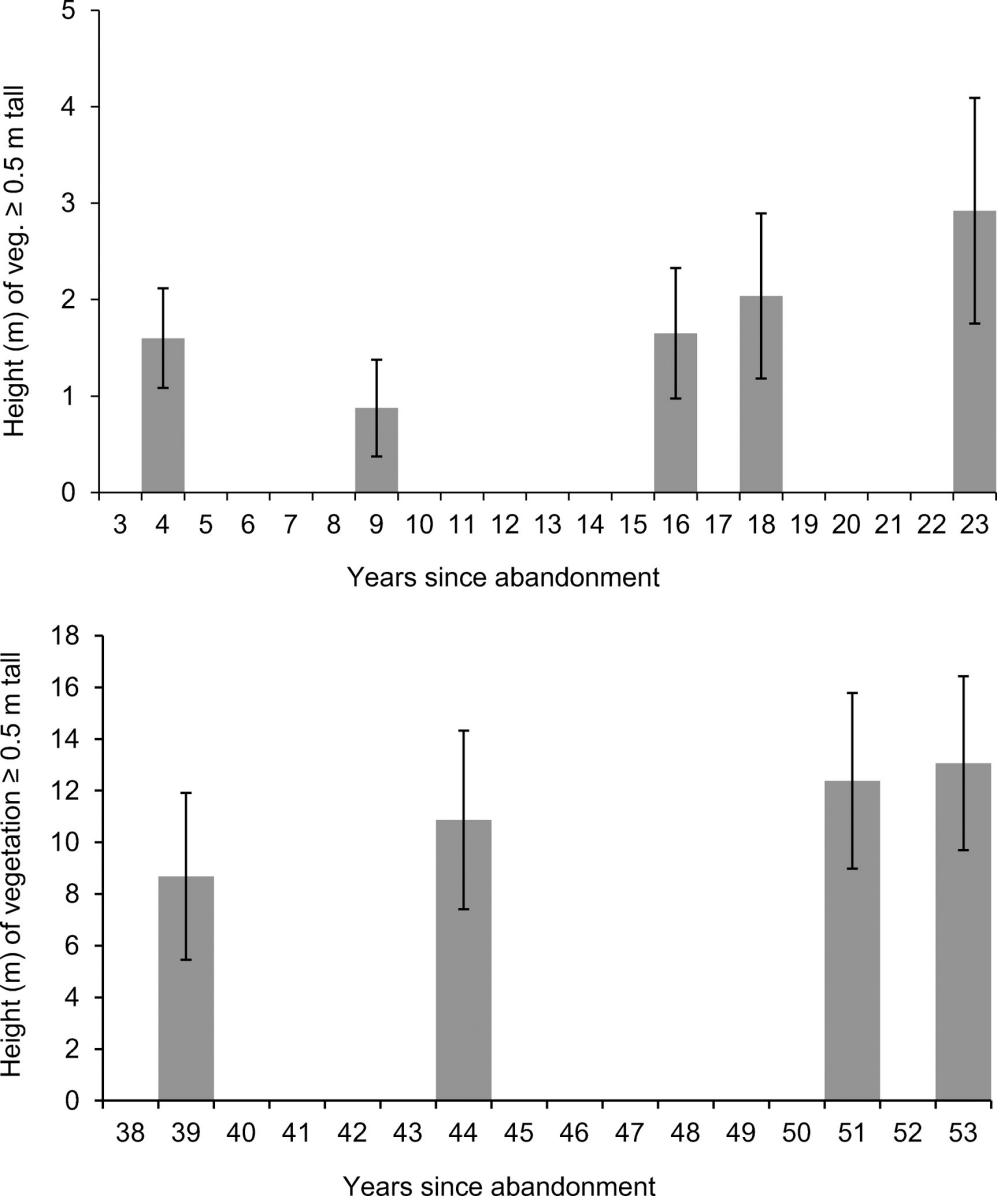
Wilderness vegetation heights. Progression of mean height (and standard deviations) of vegetation of at least 0.5 m tall in the New Wilderness (top) since abandonment in 1996, and in the Old Wilderness (bottom) since abandonment in 1961. Note the different axes between charts.

In the New Wilderness, remote sensing surveys showed that the percentage cover of woody vegetation (0.5 m or taller) increased rapidly from negligible cover four years after abandonment, to 85–86% after 18–23 years (Figs 1 and 3). The height of this woody vegetation showed a rapid increase from initial open grassland to a thicket of shrubs and young trees; after 23 years the vegetation averaged 2.9 m tall and the relatively large standard deviation indicated a very uneven canopy (Fig 4). There was an initial reduction in mean height between four and nine years post-abandonment, due to a small number of pixels representing vigorous early sucker growth of a single small grove of willow, which briefly inflated the mean height value. This small grove later became subsumed within the more widespread cover of shrub thickets that had developed by nine years post-abandonment.

In the Old Wilderness, woody vegetation taller than 0.5 m covered 97–100% of the site area in the lidar surveys over the 39–53 years post-abandonment, and so was virtually complete (Fig 2). The mean canopy height of the woody vegetation increased by 50% over the same period, to 13.1 m tall after 53 years. Little change in the standard deviation over this period, despite an increasing mean, indicated that the closing canopy was becoming relatively more uniform in height (Fig 4). Overstorey canopy closure (vegetation of 8 m or taller) already covered 57% of the Old Wilderness area by 39 years post-abandonment, increasing steadily to 91% after 53 years (Fig 2).

### Woody vegetation composition

#### Tree densities and sizes

The field survey of woody vegetation (trees and shrubs) in the New Wilderness after 24 years of abandonment found 306 trees with a minimum dbh of 3.2 cm, giving a density of 143.5 trees/ha. Few trees (5.9%) had a dbh of 10 cm or more (Table 1). Of all recorded trees, 8% were standing or leaning deadwood of a small dbh (< 10 cm), but no fallen deadwood of a recordable size was noted. This gave a live tree density of 132/ha in the New Wilderness.

**Table 1:** Tree species data.

Tree species	dbh cm	New Wilderness	Old Wilderness	Monks Wood
Common Ash	3.2–9.9	21.1	45.0	No data
	10–29.9	0.0	83.8	127.0
	≥ 30	0.0	22.4	75.5
Common Aspen	3.2–9.9	0.0	0.0	No data
	10–29.9	0.0	0.0	11.8
	≥ 30	0.0	0.0	0.0
Elm spp.	3.2–9.9	23.0	0.8	No data
	10–29.9	2.8	0.0	19.7
	≥ 30	0.0	0.0	3.0
Field Maple	3.2–9.9	0.5	3.1	No data
	10–29.9	0.0	8.5	50.3
	≥ 30	0.0	1.8	26.7
Pedunculate Oak	3.2–9.9	71.7	7.5	No data
	10–29.9	3.8	115.7	11.5
	≥ 30	0.0	80.7	24.8
Silver Birch	3.2–9.9	0.0	2.1	No data
	10–29.9	0.0	16.5	13.3
	≥ 30	0.0	1.3	2.1
Wild Service	3.2–9.9	0.0	0.0	No data
	10–29.9	0.0	0.0	0.3
	≥ 30	0.0	0.0	0.3
Willow spp.	3.2–9.9	7.0	0.0	No data
	10–29.9	1.9	0.3	5.2
	≥ 30	0.0	0.8	0.0
All live trees	3.2–9.9	123.3	58.4	No data
	10–29.9	8.2	224.7	239.1
	≥ 30	0.0	106.9	132.4
Deadwood	≥ 3.2	11.7	63.5	82.4

Densities (per ha) of live trees by size class (dbh: diameter at breast height of 1.3 m), and combined sizes of deadwood, for 2.1 ha of the New Wilderness site (24 years post-abandonment), 3.9 ha of the Old Wilderness plot (57–59 years post-abandonment) and sampling in the adjacent Monks Wood (3.3 ha combined from 33 × 0.1 ha sample plots, c. 90 years of regrowth after felling).

In the Old Wilderness, surveys after 57–59 years of abandonment recorded 1764 trees, giving a density of 453.5/ha (Table 1). Excluding the 14.0% of trees that were standing or fallen deadwood, this gave a live tree density of 390.0/ha. Most (58%) of the live trees in the Old Wilderness had a dbh of 10–29.9 cm, but more than a quarter (27%) had a dbh of 30 cm or greater (Table 1).

#### Tree species composition

Over half of all live trees in the New Wilderness were pedunculate oaks (57.3%), followed by elm (19.6%) and common ash (16.0%), with small proportions of willow (6.8%) and field maple (0.4%) (Table 1). Most willows and elms originated by suckering from adjacent shrubland. Of the standing deadwood, 80.0% was common ash and the rest was elm.

In the Old Wilderness, live trees were also dominated by pedunculate oak (52.3%), followed by common ash (38.8%), silver birch (5.1%), field maple (3.4%), willow (0.3%) and elm (0.2%). The proportion of tree species that are primarily wind-dispersed (e.g. common ash, silver birch and field maple) was much greater in the Old Wilderness (47.3% of live trees) than in the New Wilderness (16.4%).

In the Old Wilderness, combined pedunculate oak, common ash and field maple showed a 2.6% decline in live tree density between 37 and 57 years after abandonment, as trees matured and self-thinned. This was reflected in the deadwood composition at the end of this period, 57–59 years post-abandonment, which was dominated by pedunculate oak (42.9%) and common ash (27.5%), but also silver birch (27.1%). Nearly half (46.5%) of all birches were dead, compared to 0–11.8% for other species (except willow, where one of the five recorded trees was dead).

#### Shrub vegetation

**Fig 5:**
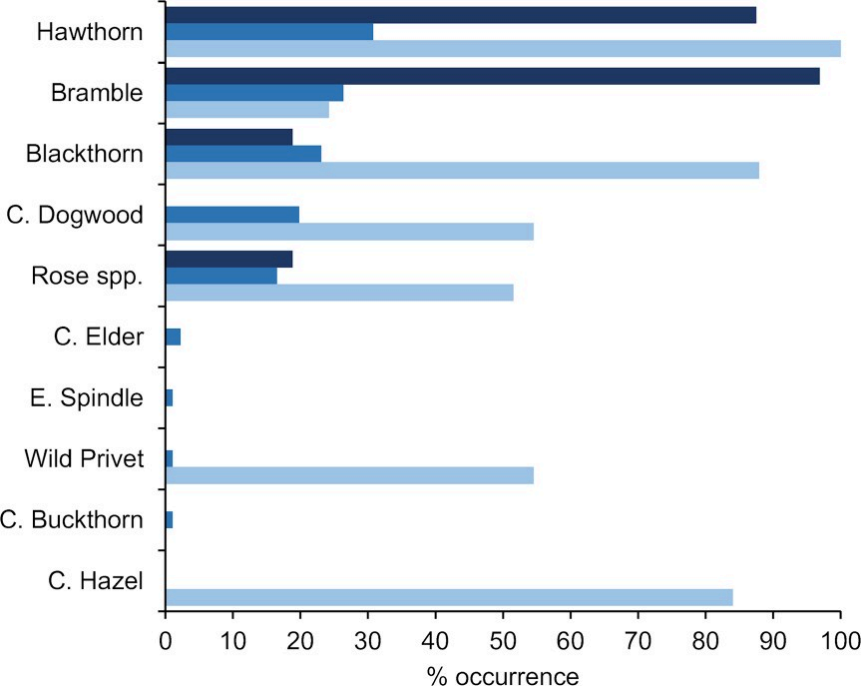
Shrub species frequency. The percentage occurrence of shrub species in sampling sites, comprising 32 quadrats of 1 m^2^ in the New Wilderness (dark blue bars), 91 quadrats of 1 m^2^ in the Old Wilderness (mid blue bars) and 33 sample plots of 0.1 ha in Monks Wood (pale blue bars).

Quadrat sampling in the New Wilderness 23 years after abandonment, and the Old Wilderness after 54 years, showed that the most frequent shrub species were hawthorn, blackthorn, rose and bramble (Fig 5). Hawthorn and bramble were particularly frequent in the New Wilderness, while common dogwood was additionally prevalent in the Old Wilderness (Fig 5).

### Patterns of tree distribution

The Moran’s I tests indicated that the distribution of all trees in both Wildernesses was non-random, with significant clustering. Tree counts per grid cell (hereafter ‘cell counts’) were spatially autocorrelated in the New Wilderness (Moran’s I = 0.26, z = 8.01, *P* < 0.01) after 24 years of abandonment, and also in the Old Wilderness (Moran’s I = 0.29, z = 10.78, *P* < 0.01) after 57–59 years.

The distance between the centre of individual grid cells in the Old Wilderness and the surrounding seed sources ranged between 0–112 m; cell counts of the number of trees were 0–27 (mean 9.4) for pedunculate oak and 0–64 (mean 6.9) for common ash. By comparison, in the New Wilderness distances from individual grid cells to seed sources were 12–122 m, and cell counts were 0–15 (mean 3.0) for pedunculate oak and 0–10 (mean 1.2) for common ash.

ZINB regression showed significant negative relationships in both Wildernesses between common ash abundance and distance from potential seed sources, with fewer ash trees at increasing distance from the adjacent Monks Wood and/or hedgerow (S2 Table). A similar relationship was found for pedunculate oak in the New Wilderness only, though the trend was less pronounced than for ash. There was no distance trend for grid cells that lacked any oaks in either Wilderness, nor ashes in the New Wilderness (S2 Table). This showed that grid cells with and without these trees were distributed broadly across the sites, reflecting a clumped distribution of trees within some grid cells but not others, regardless of distance from the seed sources.

### Landscape woodland context

Sampling surveys from 2006 in the ancient woodland of Monks Wood, adjacent to the Wilderness sites, found a density of 454/ha for trees with a dbh ≥ 10 cm, aggregated from 33 transects. Standing or fallen deadwood accounted for 18% of these trees, giving a density of 372/ha live trees of a dbh ≥ 10 cm. Comparable densities for live trees ≥ 10 cm dbh in the New Wilderness after 24 years of abandonment were 8.4/ha, and 331.8/ha for the Old Wilderness after 57–59 years. Although live tree densities in the Old Wilderness were approaching that of Monks Wood, the latter had a particularly greater density of larger trees (≥ 30 cm dbh) (Table 1).

Compared to Monks Wood, the Old Wilderness had lower densities of most tree species, but median species densities summed across comparable medium-large size classes were not significantly different (Table 1; Kruskal-Wallis test: *H* = 1.34, *P* = 0.248); however, the density of medium-large pedunculate oaks in the Old Wilderness was almost five times greater than in Monks Wood (Table 1). The species richness of trees and shrubs in the Old Wilderness (15 species) was almost as high as in Monks Wood (17 species), but the younger New Wilderness was relatively species-poor (9 species; Table 1 and Fig 5).

Several shrubs recorded in the Old Wilderness were not recorded in the Monks Wood surveys (e.g. common elder *Sambucus nigra*, spindle *Euonymus europaea*, common buckthorn *Rhamnus cathartica*), but are known to be present in small numbers (pers. obs.). Meanwhile, some species present in the Monks Wood survey had not colonised either Wilderness site (common aspen, wild service, common hazel; Fig 5).

Overall, however, compared to Monks Wood, the vegetation in both Wilderness sites was overwhelmingly dominated by pedunculate oak and thorny, berry-bearing shrubs. Pedunculate oak comprised 48% and 59% of the medium-large trees in the New and Old Wildernesses, but only 10% in Monks Wood. Among shrubs, it was only the thorny hawthorn, blackthorn, rose and bramble that all exceeded 10% frequency in the Wildernesses (except also common dogwood in the Old Wilderness; Fig 5). By contrast, just as many non-thorny or nut-bearing shrubs (such as common hazel) exceeded 10% frequency in Monks Wood.

All of the local ancient woodlands had almost total cover of woody vegetation, indicating a lack of open glades or significant tree-fall gaps. The Old Wilderness had similar woody cover from the initial lidar survey, 39 years after abandonment. At 86%, woody coverage in the New Wilderness after 23 years of growth still lagged behind these other sites.

The mean canopy height of Monks Wood in 2014 was almost 4 m taller than the Old Wilderness after 53 years post-abandonment, and 13 m taller than the New Wilderness after 23 years (Table 2). However, the mean canopy height of the Old Wilderness was within the range of other ancient woodlands in the area, and had developed an overstorey canopy extent close to that of Monks Wood and other sites, indicating that it was approaching a similar level of maturity (Table 2).

**Table 2:** Comparative tree canopy heights.

Wood	Area ha	Mean height (m)	Height sd	% cover ≥ 0.5 m	% cover ≥ 8 m
New Wilderness	2.1	2.9	1.2	86.1	0.0
Old Wilderness	3.9	13.1	3.4	99.6	90.5
Monks	139.4	16.9	4.1	99.6	96.1
Archers	15.7	17.0	4.2	99.6	96.6
Little Less	26.2	18.0	4.1	99.6	97.3
Odd Quarter	13.3	16.0	3.3	100.0	97.8
Hill	16.0	15.1	5.3	97.9	86.4
Riddy	8.2	15.0	4.5	100.0	94.8
Gamsey	4.1	14.3	4.5	100.0	91.1
Holland	26.9	18.0	5.5	99.3	94.0
Wennington	77.5	20.2	4.8	99.4	96.7
Aversley	44.8	19.8	4.3	100.0	98.9
Raveley	5.7	14.9	4.2	99.9	87.2
Lady’s	7.1	12.1	3.5	100.0	95.1

Mean canopy height and cover of woody vegetation in two Wilderness sites and in tree stands of local woodlands. Coverage by vegetation ≥ 0.5 m tall reflects all woody vegetation, and coverage ≥ 8 m tall reflects the semi-mature or mature vegetation of the overstorey tree canopy. Values for the New Wilderness derive from structure from motion data acquired by drone in 2019 (23 years post-abandonment), and other values derive from airborne lidar data acquired in 2014 (53 years post-abandonment for the Old Wilderness).

## Discussion

### Main findings

The results show that restoration and expansion of native woodland can be rapid on former farmland adjacent to existing forest. This is in line with our hypotheses, as was the finding that the tree and shrub composition on our two sites was less diverse than in the nearby ancient woodland, being dominated by species dispersed by birds and rodents (but with a minority proportion of wind-dispersed species, which was higher on the Old Wilderness with originally disturbed soil than on the New Wilderness’s grassy sward). Additionally, we found that tree colonisation had a clumped distribution, with wind-blown common ash being more abundant nearer to seed sources, but animal-dispersed pedunculate oak also showing some clustering. Despite these trees having an uneven distribution, the overall woody cover was more evenly widespread due to abundant shrub thickets.

These generalities of our study in lowland Britain are in line with other studies of woodland succession on abandoned farmland around the World, particularly in North America and Northern Europe, where land use history, ground conditions, surrounding seed sources, the availability of dispersing animals and birds, and time since abandonment all contribute to the successional trajectory of colonising vegetation [16–18, 24–26, 62]. However, our study quantifies this succession in a temperate Western European context, by detailing the spatio-temporal trends in the development of woodland structure and composition, for which data are otherwise rare and urgently needed in this region [63].

At our two Wilderness reference sites at Monks Wood, the trajectories of succession indicate that colonisation by woodland shrubs and trees begins spontaneously soon after land abandonment. A rapidly-developing shrub-thicket phase in the initial two decades after abandonment progresses to increasing tree cover, and eventually achieves almost total closed-canopy, mixed-species woodland after approximately 50 years.

The woodland vegetation developed on our sites without any intervention or management costs besides path clearance. There was no protection from browsing animals, such as brown hares *Lepus europaeus*, grey squirrels *Sciurus carolinensis*, European rabbits *Oryctolagus cuniculus*, Reeves’ muntjac *Muntiacus reevesi* and Roe deer *Capreolus capreolus*, which were variously common or abundant during some or all of the study period [51, 64, 65]. Culling limited the deer numbers in Monks Wood from the late 1990s [64], but little or no control took place in the two Wildernesses, which could have been refuges for browsing deer. Although herbivores can inhibit regeneration [17], they did not prevent rapid woodland and shrub development at our Wilderness sites.

Woodland regeneration and succession also appeared resilient to a warming climate in the region, with increasingly hot summers since the 1960s [66]. Notable droughts (rainfall deficiencies) occurred in the Monks Wood area during at least 1973, 1976, 1990, 1995 and 2003, but did not prevent the woodland cover from continuing to develop across the Wildernesses [52, 66].

### Patterns and timescales of woodland regeneration

On the New Wilderness, woody cover was advanced after just 16–18 years, with thickets of shrubs and young trees covering most (80–85%) of the site. Initially establishing as clumps of shrubs, the woody cover then became widespread, but after 23 years the New Wilderness was still in a thicket stage and yet to develop an overstorey tree canopy (8 m or taller).

In the Old Wilderness, woody vegetation cover was effectively complete by the time that remote sensing surveys began, from 39 years post-abandonment, and the overstorey tree canopy covered more than half of the site. Tree canopy closure neared completion by 53 years, and densities of medium-sized or larger trees were approaching those of neighbouring ancient woodland, though large trees were still relatively uncommon. The overstorey canopy height continued to increase steadily during 39–53 years post-abandonment, and heights for neighbouring ancient woodlands suggest that it could grow several metres taller and so was not yet fully mature.

The tree density was three times greater in the Old Wilderness after 58–59 years of colonisation compared to the New Wilderness after 23 years, perhaps reflecting greater initial establishment and/or a longer period of ongoing tree colonisation in the Old Wilderness. However, a much greater proportion of trees in the Old Wilderness were dead, and this deadwood appeared to have originated mostly from competition and self-thinning among immature trees, which was in line with other studies of woodland establishment [16, 17]. In contrast, dead trees in the New Wilderness appeared to have died from diseases, with young common ashes apparently succumbing to ash dieback *Hymenoscyphus fraxineus* (probably arriving in 2012–13) and the elms possibly to Dutch elm disease *Ophiostoma novo-ulmi* (present since the 1960s). High mortality of common ash has also occurred on another young site of natural regeneration in England [43], and will likely shape the future composition of these new woodlands as places where common ash is rare. Such mass mortality from a disease that disrupts the trajectory of natural regeneration appears to be rare in the literature [16–18].

The high proportion of dead silver birch in the Old Wilderness probably reflects typical mortality of early pioneers of this short-lived species reaching the end of their life, as well as mortality from increasingly hot summers [52, 66]. The lack of birch in the New Wilderness likely reflects its parallel decline as a local seed source in Monks Wood [52], and a greater difficulty in germinating in the original grass sward of the New Wilderness compared with the ploughed ground of the Old Wilderness. Indeed, the disturbed soil of the Old Wilderness on abandonment, and possibly the longer timescale of regeneration, was associated with a much greater proportion of wind-dispersed trees (e.g. common ash, field maple, silver birch) than in the New Wilderness.

In the Old Wilderness, although the species richness and woodland structure were converging with the ancient woodland of Monks Wood, the frequency of tree and shrub species were still rather different. Compared to Monks Wood, pedunculate oak was over-represented in the Old Wilderness and common ash was under-represented, and there was a lower frequency of most shrub species. The New Wilderness also appears likely to develop with a different composition from Monks Wood.

This discrepancy in species frequencies between long-established woodland and regenerating secondary woodland appears typical of studies elsewhere [14–18, 37, 38]. However, the history of former management in Monks Wood, and other established woodlands, may bias their composition by favouring certain species, such as historically valuable pedunculate oak and common hazel [51, 67]. This composition would be likely to differ from that of purely self-sown and naturally regenerating woodlands, such as the Wilderness plots, and this should be considered when comparing rewilded or restored sites with formerly managed woods.

Differences in local seed sources, dispersal and establishment are the fundamental principles that shape the patterns of natural regeneration in secondary woodlands [17]. An earlier assessment of the Old Wilderness found contrasting distributions between tree species within the site [38] and our results show this has persisted over several decades. Common ash trees were mainly confined to the vicinity of the surrounding woodland and hedgerows, while pedunculate oaks were more widely distributed at greater distances from seed sources. However, the frequency of oaks in the New Wilderness also declined at greater distances from Monks Wood and hedgerow trees, indicating some effect of distance decay, even over distances of only 120 m.

### Mechanisms of woodland regeneration

Common ash seeds are wind dispersed, whereas acorns of pedunculate oaks are animal-dispersed. Eurasian jays *Garrulus glandarius*, grey squirrels and wood mice *Apodemus sylvaticus* were probably the key distributors of acorns in the Wildernesses [53, 68]. These species are locally frequent or abundant [65, 69] and cache (bury) large numbers of acorns in autumn, with mice typically transporting them tens of metres before burying, whereas jays may travel hundreds of metres to the cache site [68, 70, 71].

Earlier studies of the Wildernesses found high densities of germinating acorns. After six years of abandonment, 287/ha young oaks were found on 0.9 ha of the Old Wilderness [53]. In the New Wilderness 147/ha oak seedlings were found after two years of abandonment, with just 6/ha of common ash [39]. Our results found lower densities of established oaks on both Wildernesses in later decades, presumably due to mortality. However, common ash had become more abundant on the New Wilderness, despite the original grassy sward being a potential barrier to germination.

The eventual clumped and uneven distribution of trees in both Wildernesses, indicated by the Moran’s I tests and ZINB models, likely reflects the interaction between seed source distance, dispersal mechanism and seedling mortality. Common ash tend to cluster close to parent trees, but oaks are clustered to a lesser degree, due to individual mammals or birds caching multiple acorns in close proximity [68].

The domination of thorny, berry-producing hawthorn, bramble, blackthorn and rose among the shrub thicket in the younger New Wilderness, and among Old Wilderness shrubs, also reflects seed distribution via the droppings of foraging birds, particularly berry-eating thrushes (Turdidae). The rarity of other shrubs that are frequent in the adjacent Monks Wood, such as common hazel and wild privet, is puzzling. Like acorns, hazelnuts may be expected to be distributed by animals, although in much of Britain the nuts are heavily depredated by grey squirrels before ripening [67]. The scarcity of wild privet may reflect a low frequency in the diet of berry-eating birds, or difficulty for seedlings to establish under the conditions of the Wildernesses.

The results are therefore in line with other studies demonstrating that ground conditions are important for vegetation establishment and composition in woodland restoration [16–18, 62, 72, 73]. Smaller and wind-blown seeds can germinate more easily on disturbed ground [15, 25, 39], whereas large seeds that are dispersed and buried by birds/mammals can readily establish under more varied conditions. Disturbing the ground through ploughing or scarifying could facilitate colonisation of wind-blown species. The broadly uniform establishment of thorny, berry-bearing shrubs, dispersed by songbirds, can also provide protection for tree saplings from browsing herbivores [50, 74].

Consequently, trees and shrubs that produce large seeds and berries appear to have a colonisation advantage in passive rewilding or restoration of abandoned land, at least when close to existing woodland. This advantage of animal-dispersed species, along with initial ground conditions, explains the differing compositions between the Wildernesses and the adjacent and more varied ancient woodland of Monks Wood. Similar patterns exist on regenerating wood pasture in Spain [50], early succession of English farmland [75], and on old-field regeneration in temperate North America [17]. These patterns suggest that in Western Europe, sites close to mixed seed sources containing oaks are likely to develop as young oak-dominated woodlands within 50 years of abandonment, with an initial thicket and later understorey of berry-bearing shrubs such as hawthorn, blackthorn, rose and bramble. This is particularly true of sites originally covered by a sward of grass or other vegetation. Further increases in diversity and species richness are likely to accrue over time, and more quickly for sites with disturbed ground, eventually leading to woodland plant communities of a similar species richness and functional resilience as ancient woodlands, even if the species composition may differ [14].

These overall results align with global meta-analyses, which indicate that active restoration, such as planting, appears to have negligible benefits over passive restoration of native woodland, which in many circumstances will develop over similar timescales on areas close to seed sources, with or without intervention [16, 17].

### Implications for biodiversity and other ecosystem services

The successional wooded habitats that develop during woodland restoration and passive rewilding can have significant conservation value in restoring biodiversity to degraded landscapes and damaged ecosystems [43, 63, 76]. In Western Europe, the shrub thicket stage in the initial decades after abandonment is a key habitat for several birds of conservation concern, including the willow warbler *Phylloscopus trochilus*, willow tit *Poecile montanus*, turtle dove *Streptopelia turtur* and common nightingale *Luscinia megarhynchos* [32, 77–79]. Later succession to closed-canopy, oak-dominated woodland favours other declining species, including the marsh tit *Poecile palustris* and wood warbler *Phylloscopus sibilatrix*, and large-scale afforestation benefits woodland specialists that are sensitive to habitat fragmentation [7, 48, 49, 80].

Natural disturbance processes are important components of rewilded woodlands, causing tree mortality and canopy gaps to support biodiversity [81], or maintaining a mosaic of open habitats through large herbivore browsing [43, 75]. The accumulation of significant deadwood in maturing secondary woodland, as in the Old Wilderness within 50–60 years, provides further important habitat for saproxylic species, as well as nutrient flow and carbon storage [82, 83]. Afforestation of former farmland has a greater potential for storing soil organic carbon than the cropland it replaces [84, 85], and carbon sequestration and storage can be a net benefit of large-scale woodland restoration and rewilding [86], alongside other ecosystem services such as recreation and water regulation [63, 87]. Nevertheless, passive rewilding of former agricultural land can also result in the loss of cultural traditions and landscapes and the species associated with them [10, 63]

In view of widespread farmland abandonment across Europe, and further significant areas at high risk (including in Britain), our results help to address the pressing need for targeted research to inform future landscape policy in Europe by quantifying what outcomes for ecosystem services may occur over a timescale of almost 60 years [63].

## Conclusions

The combination of field survey and remote sensing for long-term monitoring of the two Wilderness reference sites at Monks Wood, which had been undergoing passive rewilding for 24 and 59 years, provides unique direct evidence of how closed-canopy woodland re-establishes onto abandoned farmland. This native woodland restoration was approaching the structure (but not the species composition) of long-established woodlands within six decades. Such sites adjacent to ancient woodland probably reflect ideal conditions for restoration, with numerous seed sources of varied woody species close by. Nevertheless, the developing woodland was resilient to the presence of herbivores (e.g. deer, rabbits) and episodes of drought.

In the Western European context, if our results are representative, the woodland habitats that eventually develop through succession of abandoned farmland are likely to have a majority of animal-dispersed woody vegetation, depending on local seed/berry sources of trees and shrubs, and also the dispersal fauna. The presence of seed-caching and berry-eating species, particularly Eurasian jays, thrushes, wood mice and squirrels, appears essential for providing the ecosystem service of distributing shrub and tree seeds. These few species seem to provide much of the natural regeneration capability in this region, and maintaining their populations in the landscape is clearly important to achieve woodland restoration over significant areas.

Wind-blown tree seeds are also an important component of colonising vegetation, and ground disturbance may aid their establishment, which could assist with more varied woodland regeneration in shorter timescales. Plant diseases and browsing pressure from herbivores will also shape the composition of restored woodlands, although deer, rabbits and squirrels did not prevent woodland regeneration at our Wilderness sites.

Our study provides direct empirical evidence that passive rewilding has the potential to expand native woodland habitat at very low cost and within relatively short timescales, potentially generating valuable successional habitats for biodiversity. Passive rewilding can expand and buffer existing woods, as at Monks Wood, or create new woodland patches to diversify intensive farmland and act as sources for further expansion [43, 88]. As such, passive rewilding through natural regeneration could be a significant tool in afforestation policies, such as the UK’s ambition to increase woodland in England by actively planting at least 12 million trees on 185,000 ha by 2042, which has forecasted costs of £5.7 million for a ‘Northern Forest’ [89]. Incorporating targeted passive restoration into such plans could result in significant cost savings.

To better inform the use of passive rewilding and woodland restoration as a management tool, further detailed case studies of vegetation succession on former farmland are needed, particularly at larger extents and greater distances from seed sources, and on land with varying histories of intensive agriculture. Farmland abandoned more recently may have a greater legacy of high fertility and residual pesticides from longer periods of intensive agriculture, and this could influence trajectories of vegetation succession [25].

The increasing availability of remote sensing techniques enables detailed assessment of the development of woodland vegetation, including structure and species composition [42, 90]. The creation of complementary time series of remote sensing and field surveys provides a powerful basis for direct observation of woodland restoration to inform rewilding policies.

Appropriate targets for restoration outcomes are self-sustaining woodland ecosystems with natural disturbance and regeneration dynamics, and sufficient stakeholder support and public perception of wildness [19]. Habitat structure, species richness and ecosystem processes are some appropriate metrics for determining restoration success.

## Supporting information

S1 Appendix Lidar data acquisition and processing details. (DOCX)

S1 Fig (JPG)

S1 Table Summary of vegetation surveys. (DOCX)

S2 Table Results of Zero-Inflated Negative Binomial models. (DOCX)

S1 Dataset Compressed/ZIP file archive of raster ascii GIS data for Old Wilderness Canopy Height Model 2000. (ZIP)

S2 Dataset New Wilderness vegetation survey data (tree counts and shrub presence). (XLSX)

S3 Dataset Old Wilderness vegetation survey data (tree counts and shrub presence). (XLSX)

S4 Dataset Monks Wood vegetation survey data (tree counts and shrub presence). (XLSX)
