# Supplementary material for: Long-term woodland restoration on lowland farmland through passive rewilding
Source: PLoS One. 2021 Jun 16;16(6):e0252466. doi: 10.1371/journal.pone.0252466 (PMC8208563; doi:10.1371/journal.pone.0252466)
Supplement: S1 Appendix — (DOCX) [file pone.0252466.s001.docx]

S1 Appendix. Details of lidar data acquisition and processing

The lidar datasets for this study were collected in leaf-on conditions during four separate years: 2000, 2005, 2012 and 2014. In all cases, the lidar sensor was mounted on a fixed-wing aircraft flown at altitudes between 1000 and 1600 m. The lidar scanners used differed considerably in their acquisition parameters, especially in the number of returns they were able to record per submitted pulse (Table S1): while each pulse return always had the information on the 3D geographical coordinates (i.e. x, y, z) of the surface from which it was reflected, and the strength of the return signal (i.e. intensity), information on whether it was the first, second, third or fourth return of the emitted laser pulse (i.e. the return number) was lacking in the older scanners. Therefore, only the echo categories “first of many” and “only” were used, because they represent surface hits and were attainable from every lidar dataset.

The lidar echoes of all datasets were classified into ground or vegetation hits following the method of Axelsson (2000), as implemented in LAStools software. Next, the lidar returns were scaled to ‘above ground level’ by subtracting a Digital Terrain Model (DTM) from their elevation values (z-coordinates). The DTM used in this process originated from leaf-off lidar data collected in winter of 2014. This DTM was used because lidar data collected during leaf-off conditions contains more accurate 3D description of the terrain. The DTM had a 1 m spatial resolution and it was interpolated from classified ground hits using inverse distance weighted interpolation (IDW).

The scaled data were then used to create Canopy Height Models (CHM). A CHM is a raster representation of the top canopy and it is created from the first lidar returns, the surface returns (as opposed to DTMs that are created from ground returns only). The CHMs had 1 m spatial resolution and they were created by local maxima search within a defined maximum radius. That is, each raster cell was assigned the maximum height value of the lidar return closest to it. The maximum radius used in this search was adjusted between the different lidar datasets so that the final cell size of the raster CHM was adequate in relation to the lidar dataset’s pulse density (Table S1). The CHMs gave detailed and comparable information about the development of the vegetation during the 15 year period.

Table S1. Acquisition parameters of the different lidar datasets used in the study

| **Acquisition parameter** | **Year** | | | | |
| --- | --- | --- | --- | --- | --- |
|  | **2000** | **2005** | **2012** | **2014** | **2014** |
| Scanner | Optech ALTM-2010 | Optech ALTM-3033 | Leica ALS50-II | Leica ALS50-II | Leica ALS50-II |
| Wavelength | 1047 nm | 1064 nm | 1064 nm | 1064 nm | 1064 nm |
| Flying altitude (agl) | 1000 m | 2100 m | 1600 m | 1600 m | 1600 m |
| Flying date | 10/06/2000 | 26/06/2005 | 15/09/2012 | 01/06/2014 | 16/03/2014 |
| Max. No. of registered returns per pulse | 2 | 2 | 4 | 4 | 4 |
| Average pulse density* | c. 0.2/m^2^ | 0.5/m^2^ | c. 2.7/m^2^ | c. 2.7/m^2^ | c. 2.7/m^2^ |
| **Nominal average pulse density across the study area. As the scanners differ in the number of returns they record, the density parameter refers to the density of returns categorised as 'first of many' or 'only', i.e. surface hits* | | | | | |

Reference

Axelsson P. DEM generation from laser scanner data using adaptive TIN models. Int. Arch. Photogramm. Remote Sens. 2000; 33: 110-117.

Data availability

Original CHM datasets are available separately as additional supplementary information. The raw unprocessed lidar data is available from the Natural Environment Research Council's Data Repository for Atmospheric Science and Earth Observation (CEDA) at the following locations:

**2005 leaf-on:** http://data.ceda.ac.uk/neodc/arsf/2005/02_19

**2012 leaf-on:** http://data.ceda.ac.uk/neodc/arsf/2012/GB12_05/GB12_05-2012_259_Monks_Wood

**2014 leaf-on:** http://data.ceda.ac.uk/neodc/arsf/2014/GB12_05/GB12_05-2014_152a_Monks_Wood

**2014 leaf-off:** http://data.ceda.ac.uk/neodc/arsf/2014/GB12_05/GB12_05-2014_075_Monks_Wood
