# Supplementary figures and images for: Long-term woodland restoration on lowland farmland through passive rewilding
Source: PLoS One. 2021 Jun 16;16(6):e0252466. doi: 10.1371/journal.pone.0252466 (PMC8208563; doi:10.1371/journal.pone.0252466)

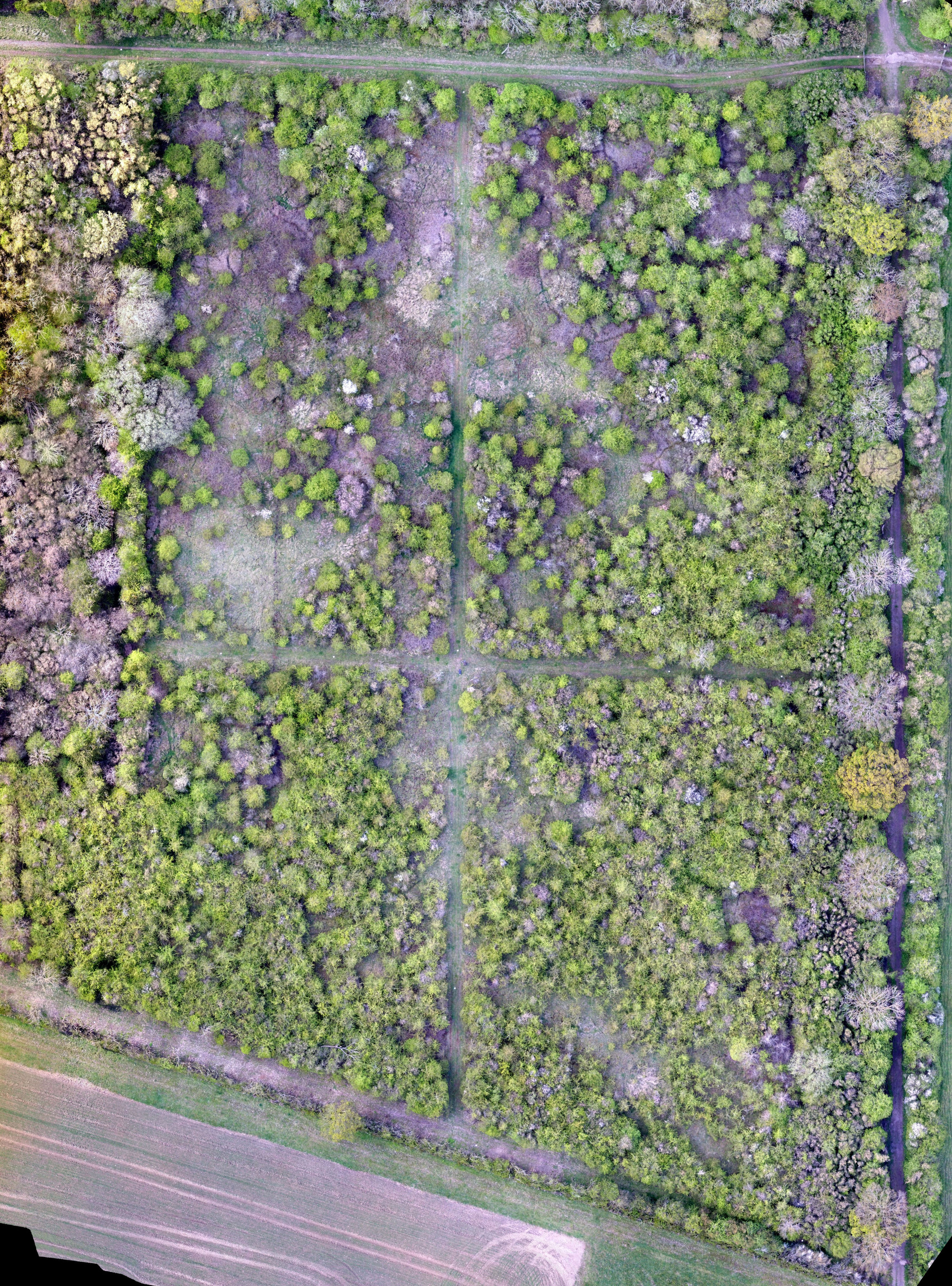

Supplement: S1 Fig — (JPG) [file pone.0252466.s002.jpg]
