# Supplementary material for: Long-term woodland restoration on lowland farmland through passive rewilding
Source: PLoS One. 2021 Jun 16;16(6):e0252466. doi: 10.1371/journal.pone.0252466 (PMC8208563; doi:10.1371/journal.pone.0252466)
Supplement: S1 Table — (DOCX) [file pone.0252466.s003.docx]

S1 Table. Summary of vegetation surveys. The extent, year, types and sample sizes (*n* = number of locations) of vegetation surveys carried out at each study site. Tree species, size & density surveys, and shrub species frequency surveys, involved field surveying. Lidar and structure-from-motion were remote sensing surveys.

|  | New Wilderness | | | Old Wilderness | | | Monks Wood | | | Local ancient woodlands | | |
| --- | --- | --- | --- | --- | --- | --- | --- | --- | --- | --- | --- | --- |
| Survey type | Year | Extent | *n* | Year | Extent | *n* | Year | Extent | *n* | Year | Extent | *n* |
| Tree species, size & density | 2020 | Whole site; DBH ≥ 3.2 cm | 1 | 2002, 2008, 2013, 2018-2020 | Whole site; DBH ≥ 3.2 cm | 1 | 2006 | 100 m x 10 m transects; DBH ≥ 10 cm | 33 | No data | No data | No data |
| Shrub species frequency | 2019 | 1 m^2^ quadrat sampling | 32 | 2015 | 1 m^2^ quadrat sampling | 91 | 2006 | 100 m x 10 m transects | 33 | No data | No data | No data |
| Lidar | 2000, 2005, 2012, 2014 | Whole site | 1 | 2000, 2005, 2012, 2014 | Whole site | 1 | 2014 | Whole site | 1 | 2014 | Whole site | 11 |
| Structure-from-motion | 2019 | Whole site | 1 | No data | No data | No data | No data | No data | No data | No data | No data | No data |
