# Supplementary material for: Long-term woodland restoration on lowland farmland through passive rewilding
Source: PLoS One. 2021 Jun 16;16(6):e0252466. doi: 10.1371/journal.pone.0252466 (PMC8208563; doi:10.1371/journal.pone.0252466)
Supplement: S2 Table — (DOCX) [file pone.0252466.s004.docx]

Table S2. Results of Zero-Inflated Negative Binomial models showing the relationship (count models) between the number of Common Ash or Pedunculate Oak trees in grid cells (response variables) and the nearest distance of cells to seed sources in the forest edge or mature trees (covariate) in the Old Wilderness (ash: theta = 0.60, log-likelihood = -209.1; oak: theta = 2.93, log-likelihood = -299.8) and New Wilderness (ash: theta = 2.68, log-likelihood = -66.8; oak: theta = 3.05, log-likelihood = -111.1). The relationship between the occurrence of zero counts (no trees) and distance is shown in the zero-inflated models. Bold font denotes significant relationships.

|  | New Wilderness | | | |  | Old Wilderness | | | |
| --- | --- | --- | --- | --- | --- | --- | --- | --- | --- |
| Variable | Estimate | SE | CI 25-75 % | |  | Estimate | SE | CI 25-75 % | |
| *Number of Common Ashes* | | |  |  |  |  |  |  |  |
| Count model | | | |  |  |  |  |  |  |
| Intercept | 1.94 | 0.35 | 1.25 | 2.62 |  | 3.54 | 0.42 | 2.71 | 4.37 |
| Distance (m) | **-0.03** | **0.01** | **-0.04** | **-0.01** |  | **-0.05** | **0.01** | **-0.07** | **-0.02** |
| Log (theta) | 0.98 | 1.22 | - | - |  | -0.50 | 0.22 | - | - |
| Zero-inflation model | |  |  |  |  |  |  |  |  |
| Intercept | -0.23 | 0.69 | 1.59 | 1.13 |  | -8.05 | 3.88 | -15.66 | -0.45 |
| Distance (m) | 0.01 | 0.01 | -0.02 | 0.04 |  | **0.12** | **0.05** | **0.02** | **0.23** |
| *Number of Pedunculate Oaks* | | |  |  |  |  |  |  |  |
| Count model |  |  |  |  |  |  |  |  |  |
| Intercept | 1.91 | 0.21 | 1.50 | 2.31 |  | 2.43 | 0.14 | 2.17 | 2.70 |
| Distance (m) | **-0.02** | **0.00** | **-0.03** | **-0.01** |  | 0.00 | 0.00 | -0.01 | 0.00 |
| Log (theta) | 1.11 | 0.58 | - | - |  | 1.07 | 0.21 | - | - |
| Zero-inflation model | |  |  |  |  |  |  |  |  |
| Intercept | -3.81 | 2.11 | -7.94 | 0.32 |  | -0.81 | 0.80 | -2.39 | 0.76 |
| Distance (m) | 0.02 | 0.02 | -0.02 | 0.06 |  | -0.09 | 0.05 | -0.17 | 0.00 |
